# LIVING WITH MULTIMORBIDITY AND FINANCIAL TOXICITY: A PATIENT- AND CLINICIAN-INFORMED FRAMEWORK

**DOI:** 10.1093/geroni/igae098.3096

**Published:** 2024-12-31

**Authors:** Cara McDermott, Michael Lourie, Hudson Hazlewood, Caroline Sloan

**Affiliations:** Duke University, Durham, North Carolina, United States; Duke University, Durham, North Carolina, United States; Duke University, Durham, North Carolina, United States; Duke University, Durham, North Carolina, United States

## Abstract

Most older adults in the US have multimorbidity (2+ serious illnesses). Living with multimorbidity impacts patient quality of life, specifically managing the symptoms of multiple diseases and accompanying medications, emotional strain, and financial toxicity, or the financial stress of having unaffordable medical expenses. We developed an initial framework describing financial toxicity in multimorbidity to inform a financial toxicity screening tool and associated interventions for the clinical setting. We then iteratively refined the framework with patient and clinician feedback. We recruited 8 patients with multimorbidity taking 5+ medications from Duke Primary Care, along with 8 clinicians. We conducted semi-structured interviews with patients to describe their experiences living with multimorbidity and financing/prioritizing healthcare needs. In focus groups with clinicians, we explored ways to adapt existing financial toxicity screening questionnaires plus barriers/facilitators to future implementation of financial toxicity interventions in primary care clinics. Our initial framework included the dynamic nature between acute stressors (e.g., heart failure exacerbation), financial coping (e.g., borrowing money), financial reserve (e.g., insurance coverage), and patient outcomes (e.g., quality of life). We added/clarified the following domains following patient interviews and clinician focus groups: caregiver support/involvement (e.g. help with bills), health system constraints (e.g. cannot waive copays), health system-based support (e.g. charity care), community-level supports/programs (e.g. food banks), clinician moral distress (e.g. anger at not being able to provide optimal care), clinician financial coping (e.g. changing prescription based on cost). Next steps include using the framework to adapt screening questionnaires and interventions to detect and address financial toxicity in multimorbidity.

